# Ultrasound-Guided Localization of the Refill Port for Intrathecal Infusion Pump Recharge: A Systematic Review

**DOI:** 10.3390/jcm14207178

**Published:** 2025-10-11

**Authors:** Beatriz Lechuga Carrasco, Nicolás Cordero Tous, Andrés Reinoso-Cobo, Jonathan Cortés-Martín, Juan Carlos Sánchez-García, Raquel Rodríguez-Blanque, Rafael Gálvez Mateos

**Affiliations:** 1 Virgen de las Nieves, University Hospital, 18014 Granada, Spain; beatrizlcarrasco@gmail.com (B.L.C.); nicolas.cordero.sspa@juntadeandalucia.es (N.C.T.); rafaelgalvez@hotmail.com (R.G.M.); 2 Department of Nursing and Podiatry, Faculty of Health Sciences, University of Malaga—Teatinos, Arquitecto Francisco Peñalosa 3, 29071 Malaga, Spain; andreicob@uma.es; 3 Department of Nursing, Faculty of Health Sciences, University of Granada, 18071 Granada, Spain; jsangar@ugr.es (J.C.S.-G.); rarobladoc@ugr.es (R.R.-B.)

**Keywords:** intrathecal infusion, ultrasound, refill port, pain

## Abstract

**Background:** Managing pain with intrathecal infusion pumps has significantly improved the treatment of individuals whose pain is uncontrollable by other methods. Using ultrasound to locate the refill port of these infusion pumps may offer an improvement over traditional methods. **Objective:** The objective of this systematic review is to update existing knowledge on the use of ultrasound for locating the refill port in intrathecal infusion pumps. **Methods:** The PRISMA review protocol was followed, and the review was registered in PROSPERO under registration number CRD 42024595671. **Results:** The main findings indicate that this technique is primarily used only in complex cases where access is difficult. Pain assessment, patient satisfaction, and recharge time compared to the traditional method are crucial factors for selecting the type of process to implement. **Conclusions:** No conclusive data are presented regarding the technique’s effect on pain reduction, patient satisfaction, reduction in time spent refilling the pump, or the prior experience level of the professional performing it, but notable improvements in these aspects are observed in certain situations.

## 1. Introduction

### 1.1. Background

Pain is an unpleasant sensory and emotional experience associated with, or resembling that associated with, actual or potential tissue damage [1]. It is prevalent in numerous clinical settings across various specialties, which leads to a significant demand for medical care. However, pain management is often limited. Among these limitations is the difficulty in measuring pain, as it is an experience strongly influenced by subjectivity.

Many types of pain exist. Patients who have intrathecal infusion pumps typically suffer from chronic pain that has not responded to other treatment modalities. These pumps are also used in oncological patients or those requiring palliative care.

Intrathecal infusion devices are an excellent method for treating individuals with refractory chronic pain, as stated by Rey-Ares et al. [2]. Patients report an improved quality of life due to the use of these devices, as shown by Narváez Sarmiento et al. in their study [3]. Recharge intervals typically range from one to three months, as indicated in the report by Karri J et al. [4], depending on the dose and concentration of the analgesic drug [5].

Traditionally, a template provided in refill kits was used to recharge intrathecal infusion pumps. These plastic templates simulate the pump’s size and feature a central hole that, when correctly positioned on the patient’s abdomen, should align with the refill port. This method is prone to localization errors, especially in overweight patients with thickened abdominal walls due to lipid deposits. Patients undergoing this technique sometimes report discomfort associated with repeated needle sticks to locate the refill port. More severe and potentially life-threatening complications can also occur, such as depositing high-concentration drugs outside the pump, as argued by Grape S et al. [6].

The use of ultrasound has expanded to a multitude of healthcare interventions. It is employed in the management of pregnant women, for ultrasound-guided infiltrations, and for screening and diagnosing certain cardiac and digestive pathologies, and increasingly for critical care patient management. Notably, it is a harmless and easy-to-use method. When using ultrasound for a refill, the refill port is first located with the ultrasound device. Since the port is a silicone structure, it appears as a vertical anechoic area on the screen. The pump itself is metal, so it will look hyperechoic. The surrounding tissues, such as muscle fibers, produce few return echoes and create a hypoechoic image.

The pump is located by palpation, and with a skin marker, the refill port is marked using the ultrasound. In more complex cases, such as when the device is not well-anchored to the internal tissue, the ultrasound can be used throughout the refill procedure with a sterile cover. Potential drawbacks of this technique include access to an ultrasound machine and the experience level of the professionals [2].

However, ultrasound devices are typically available within pain units, so they do not represent an extra cost.

By locating the exact position of the refill port, it is possible to access the reservoir with a single puncture. Regarding complications, ultrasound is harmless and does not increase the risks associated with the technique. As mentioned, in some cases, it can actually reduce complications by preventing multiple punctures or changes in the needle’s angle within the skin [4].

This technique can reduce recharge time and enhance patient safety. This could lead to increased satisfaction for both the professionals performing the procedure and the patients receiving care [3].

### 1.2. Objective

Given the above, the objective of this systematic review is to update existing knowledge on the use of ultrasound for locating the refill port of intrathecal infusion pumps.

## 2. Methodology

The methodology employed for this report was a systematic review of the scientific literature published on the ultrasound-guided refill of intrathecal infusion pumps. This review followed the Preferred Reporting Items for Systematic reviews and Meta-Analyses (PRISMA) protocol, which consists of a 27-item checklist covering the most representative sections of an original article, along with guidelines for their development. This systematic review was conducted according to a predefined protocol, available at http://www.crd.york.ac.uk/PROSPERO/ with the registration number CRD 42024595671, accessed on 11 September 2025.

### 2.1. Eligibility Criteria

We selected randomized clinical trials (RCTs), descriptive studies, and case reports—including those from both living and deceased subjects, if they belonged to preclinical feasibility studies and provided relevant data for this systematic review. All were published within the last twenty years (2004–2024). This broad timeframe was chosen because the topic under study is uncommon, and the goal was to compile all available published information. Articles had to provide information on the refill of intrathecal infusion pumps using ultrasound, with no restrictions on the language of publication.

### 2.2. Information Sources

The literature search was conducted in the following databases: Scopus, PubMed, CINAHL, SciELO, and Cochrane Library. A manual search was also performed by examining the reference lists of identified studies to find other relevant articles.

The structured language used for the search was derived from MeSH terms and Health Sciences Descriptors (DeCS). The English descriptors used were: “ultrasound,” “ultrasound-guided,” “refill,” “refill port,” “pocket fill,” “pump refill,” “intrathecal,” “intrathecal pump,” and “intrathecal pumps.” The Boolean operators employed were “OR” and “AND.”

### 2.3. Search Strategy

Table 1 Below details the search strategy used for this work, along with the date the search was conducted.

### 2.4. Data Extraction Process

After executing the search strategy, the identified articles were transferred to the Mendeley web application using the Mendeley Web Importer tool. They were then organized into folders according to the database from which they were obtained, and all duplicates were removed.

The included studies consisted of Randomized Controlled Trials (RCTs) and case reports whose objectives were to evaluate the use of ultrasound for refilling intrathecal infusion pumps, and which were published between 2004 and 2024. Two reviewers (JCM and BLC) independently screened the title, abstract, and keywords of each identified study from the search and applied the inclusion and exclusion criteria. For potentially eligible studies, the same procedure was applied to the full-text articles. Discrepancies between the reviewers were resolved through discussion or by a third reviewer (RGM).

Data regarding quality, patient characteristics, interventions, and relevant outcomes were extracted independently by two reviewers (JCM and BLC).

### 2.5. Data Collection Process and Collected Data

Two reviewers (JCM and BLC) extracted the notable data from each included article. They also assessed the strengths and weaknesses of each RCT and case report.

The article selection process is explained in more detail in the Section 3.

### 2.6. Risk of Bias in Individual Studies

To perform the methodological evaluation of the selected articles for this study, we analyzed the design, methodology, and study type of each work, aiming to select the most specific methodological evaluation scale for each case.

Of the 13 articles, 9 were case studies and 4 were RCTs.

To evaluate the methodological quality and risk of bias of the included case reports, the JBI Critical Appraisal Checklist for Case Reports was used. This tool, developed by the Joanna Briggs Institute, allows for a systematic and qualitative assessment of each study, ensuring a rigorous and transparent evaluation of the evidence. The results of this appraisal are presented in the following table.

Table 2 below shows the results obtained after the methodological evaluation using the JBI scale.

For articles whose methodology corresponded to Randomized Clinical Trials (RCTs), the scientific quality was assessed using the PEDro scale. This scale provides information on clinical scientific evidence and scores it based on indicators, adding 1 point for each present indicator and 0 points if absent, allowing for a total score of 10 points. If a clinical trial obtains a score between 9 and 10, it indicates very good quality; between 6 and 8 indicates good quality; between 4 and 5 indicates fair quality; and a score below 4 indicates poor quality. In the case of the articles selected for this systematic review, the values ranged from 5 to 9, consequently receiving an average score of 6.5, which indicates that the average scientific quality is considered “good quality.” The quality evaluations for each of the RCTs can be observed in Table 1.

The results obtained after the methodological evaluation using the PEDro scale are shown in the following table (Table 3).

In addition, to evaluate the risk of bias of the included randomized clinical trials (RCTs), the RoB 2 (Risk of Bias 2.0) tool was used. This instrument, developed by the Cochrane group, assesses methodological quality across five key domains: randomization, deviations from intended interventions, missing data, measurement of the outcome, and selection of the reported result. The risk of bias judgments (low, some concerns, or high) for each domain are detailed in the following table (Table 4).

Based on the information provided by this review, a series of premises are derived that will serve to standardize concepts regarding the use of ultrasound for refilling intrathecal infusion pumps. The scarcity of studies on this type of technique is confirmed.

## 3. Results

**Figure 1:**
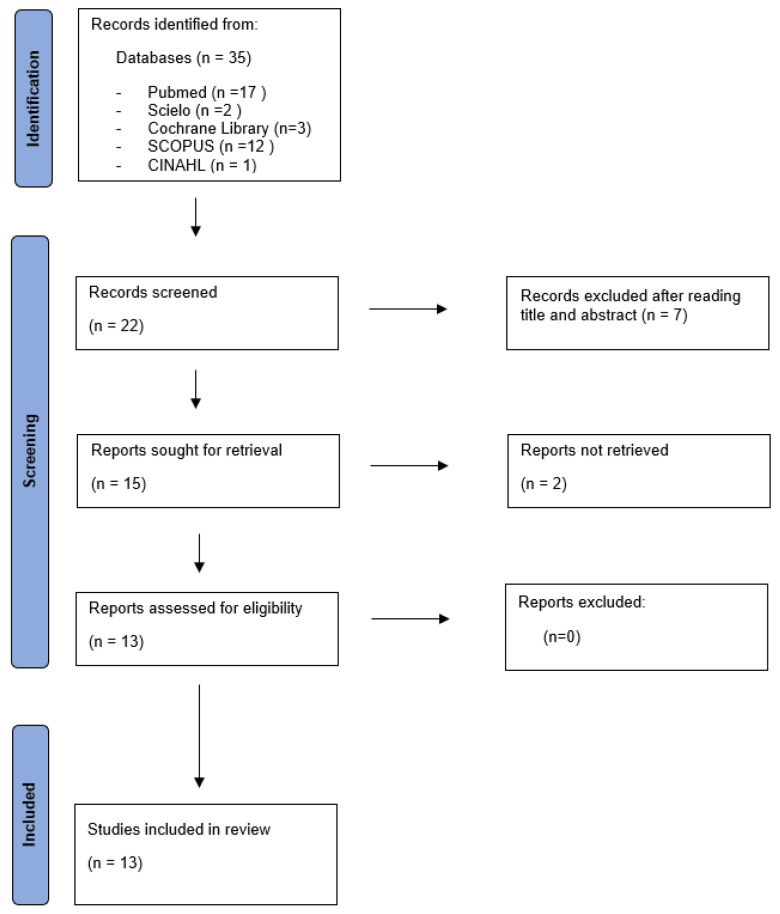
Flow diagram.

The flowchart of this systematic review is presented below (Figure 1).

The following table (Table 5) presents a summary of the main results.

**Figure 2:**
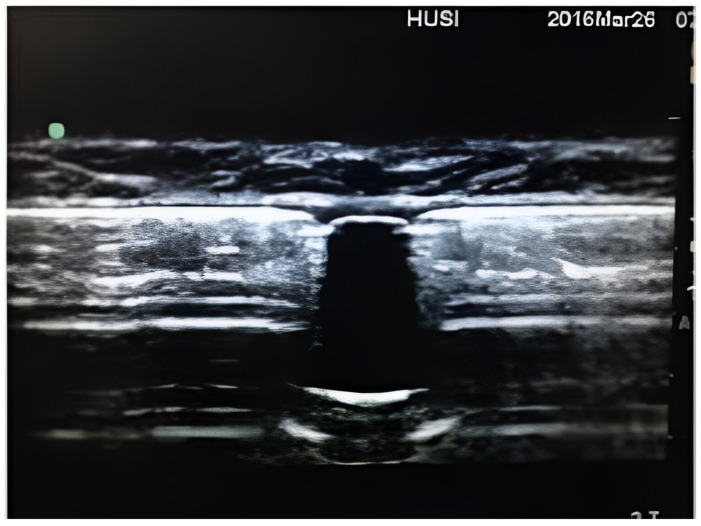
Refill port as seen with the ultrasound device.

In Figure 2, a refill port visualized by ultrasound can be observed. The first case found was published in 2007 by Hurdle et al. [7], highlighting the potential relevance of using ultrasound to locate the refill port for intrathecal infusion pumps in difficult cases. They emphasize that the technique is simple and effective, although they could not provide reliable data on a reduction in procedure time. Similarly, Shankar et al. [8] discussed the use of ultrasound in a challenging case, noting its effectiveness and its role in helping to prevent complications. Peccora et al. [10] described a case where ultrasound was used to detect fluid extravasation into the subcutaneous pocket during refilling, underscoring its importance for detecting such issues and preventing potential complications.

Furthermore, Maneyapanda et al. [12] also emphasized the use of ultrasound in difficult cases, presenting three clinical examples with challenging access and observing that ultrasound could prevent complications. In their work, Caruso et al. [13] reported two clinical cases with patients who had difficult access to the refill port, suggesting that ultrasound could be useful in these situations but that more in-depth study is needed. Consistent with this, García Eslava et al. [14] presented another case where ultrasound was used to locate the refill port, emphasizing that it minimizes risks and the number of punctures. They also highlighted the short training time—approximately 30 min—required for inexperienced personnel to learn the technique. Finally, Pinho et al. [15] also studied a case of difficult access, highlighting the use of the indirect method by marking the area on the patient’s abdomen with the aid of ultrasound. They noted that ultrasound is a simple and safe method that can prevent complications.

Other research has focused on preclinical studies. Gofeld et al. [9] conducted a study with cadavers, determining a 100% predictive value for needle placement. Consistent with García Eslava et al. [14], they emphasized the ease with which inexperienced professionals can learn the technique. They also provided a differentiated sonographic description of intrathecal infusion pumps. Delving deeper into this topic, Saulino et al.’s [11] descriptive study examined intrathecal infusion pumps ex vivo using ultrasound. They highlighted a rapid learning curve for the technique and noted that these devices possess unique sonographic characteristics.

Lastly, Maino et al. [16] conducted a 24-month study comparing intrathecal infusion pump refilling using ultrasound versus the traditional method. Their results suggest that the traditional method requires fewer punctures for refilling. In a separate study with 22 patients, Matthys et al. [17] found that the use of ultrasound prevents complications and is more effective in patients where the distance from the pump to the abdominal surface is greater than 10 mm. However, they found no difference in patients where the distance is less than 10 mm. Similarly, Singa et al. [18] conducted a study with 107 patients to assess their preferences for refilling (ultrasound vs. traditional method). On average, patients preferred ultrasound-guided refills because they found them to be less painful. However, they did not believe these refills were faster than the traditional method. To conclude, the most recent study by Stone et al. [19] used a sample of 17 patients to determine satisfaction with the technique, assessing pain, patient satisfaction, and procedure duration. Their conclusions showed that needle-in-body time was reduced, although not significantly. They also found no improvements in terms of pain perception, patient satisfaction, or professional-perceived difficulty.

## 4. Discussion

This systematic review aimed to update the existing knowledge on the use of ultrasound for locating the refill port of intrathecal infusion pumps. The topic has a limited number of publications, highlighting its specificity and the pioneering nature of this report.

Regarding the technique’s efficacy, there is a broad consensus favoring the use of ultrasound over the traditional method, which uses a template or is performed “blindly,” as presented in the study by Matthys et al. [17]. However, some articles suggest no significant difference between the two techniques, noting that ultrasound is often reserved for more complex situations. These include cases of seroma accumulation around the pump or when pump access is difficult due to patient-specific factors, as described by Peccora et al. [10]. Matthys et al. [17] also mention that ultrasound is more effective in patients where the distance from the skin to the pump surface is greater than 10 mm.

Most of the reviewed articles provide limited information on the technique’s safety. While some indirectly mention that complications can be reduced, no measurable data are presented in this regard, according to Narváez Sarmiento et al. [3].

In articles analyzing patient pain during the procedure, a significant decrease or absence of pain is established with the use of ultrasound compared to the traditional method, according to Singa et al. [18]. However, some reports, such as that by Stone et al. [19], note no difference in pain reduction.

Concerning the procedure duration, no significant differences are observed between the two techniques. Some reports suggest that ultrasound may increase procedure time, while others state that it decreases the time, though not significantly (Caruso et al. [13]). This discrepancy suggests that procedure time would be an interesting topic for future investigation to ascertain if it is genuinely shortened with either technique.

Generally, most articles reviewed use ultrasound for cases with difficult refill port access, and the majority are case reports with only one to three patients, as stated by Hurdle et al. [7]. Studies with larger patient samples do not mention cases where refill port access was more complex.

There is little emphasis on the professional role of those performing the technique, with the assumption that they are all physicians. The performance of this technique by nursing professionals is not mentioned. However, García Eslava et al. [14] mention that the average learning time for ultrasound-guided intrathecal pump refilling is no more than 30 min.

The main limitation of this review was the scarcity of publications on the topic. Furthermore, most cases are case reports with few patients and lack validated assessment scales.

Future research directions derived from this study should focus on using validated scales and measuring relevant data such as pain during the technique, patient satisfaction, time spent on refilling, and the learning curve for professionals. Similarly, it would be beneficial to explore the professional roles that carry out the technique, as nurses perform this procedure in many pain units, and their approach warrants investigation.

## 5. Conclusions

Patients with intrathecal infusion pumps require frequent pump refills. This technique has traditionally been performed using a plastic template or “blindly.” However, in some cases, the use of ultrasound has been shown to potentially facilitate the technique, especially in cases of greater access complexity.

It has been observed that using an ultrasound device can be accessible to professionals without extensive training in its operation, which makes the technique easier and more approachable for both professionals and patients. The learning curve for this technique could be short, which would facilitate the training of the professionals involved.

No conclusive data are presented regarding the technique’s effect on pain reduction, patient satisfaction, reduction in time spent refilling the pump, or the prior experience level of the professional performing it. This area could be interesting for future studies, including validated scales and questionnaires to shed light on these types of data.

Finally, the role of nursing professionals as potential individuals responsible for refills should be highlighted.

## Figures and Tables

**Table 1:** Search string.

Database	Search String
Scopus	(ultrasound OR ultrasound-guided) AND (refill OR refill AND port OR pocket AND fill OR pump AND refill) AND (intrathecal OR intrathecal AND pump)
Pubmed	(ultrasound OR ultrasound-guided) AND (refill OR refill port OR pocket fill OR pump refill) AND (intrathecal OR intrathecal pump OR intrathecal pumps)
Scielo	intrathecal AND refill AND ultrasound
Cinahl	intrathecal AND refill AND ultrasound
Cochrane Library	Intrathecal AND refill AND ultrasound

**Table 2:** Methodological Evaluation According to JBI.

Author	Patient Characteristics	Patient History as a Timeline	Clear Clinical Condition	Clear Diagnostic Tests	Clear Intervention	Clear Post-Intervention Condition	Adverse/Unforeseen Events	Key Lessons
Hurdle et al. [7]	YES	YES	YES	YES	YES	YES	NO	YES
Shankar et al. [8]	YES	YES	YES	YES	YES	YES	NO	YES
Gofeld et al. [9]	NO	NO	YES	YES	YES	NO	NO	YES
Peccora et al. [10]	YES	NO	YES	YES	YES	YES	YES	YES
Saulino et al. [11]	YES	YES	YES	YES	YES	YES	NO	YES
Maneyapanda et al. [12]	YES	YES	YES	YES	YES	YES	NO	YES
Caruso et al. [13]	YES	YES	YES	YES	YES	YES	NO	YES
García Eslava et al. [14]	YES	NO	YES	NO	YES	YES	NO	YES
Pinho et al. [15]	YES	YES	YES	YES	YES	YES	NO	YES

**Table 3:** Methodological Evaluation According to PEDro.

Author	Article	**Score**
Maino et al. [16]	Ease of Fill Port Access During the Ultrasound-Guided vs. the Blind Refill Technique of Intrathecal Drug Delivery Systems With a Raised Septum, a Prospective Comparison Study	**7**
Matthys et al. [17]	Accuracy of Template Versus Ultrasound Identification of the Reservoir Access Port of Intrathecal Drug Delivery System	**5**
Singa et al. [18]	A Comparison of Refill Procedures and Patient Outcomes Following Ultrasound-Guided and Template-Guided Intrathecal Drug Delivery Systems With Recessed Ports	**7**
Stone et al. [19]	Ultrasound guidance versus landmark guidance for intrathecal baclofen pump refill: A randomized pilot study	**7**

**Table 4:** Methodological Evaluation According to Rob 2.

Author	D1	D2	D3	D4	D5	Overall
Maino et al. [16]						
Matthys et al. [17]						
Singa et al. [18]						
Stone et al. [19]						

D1. Randomization Process. D2. Deviations from Intended Interventions. D3. Missing Outcome Data. D4. Measurement of the Outcome. D5. Selection of the Reported Result. Green. Low Risk. Yellow. Some concerns. Red. High risk.

**Table 5:** Results table.

Author/Year	Design/Sample	Objective	Result	Conclusion
Stone et al., 2023 [19]	Randomized controlled trial. 17 patients. Period of 44 months.	To determine the feasibility of using ultrasound in the refill of intrathecal infusion pumps in difficult cases for improving patient satisfaction.	17 patients underwent 21 refills (12 template-guided and 9 ultrasound-guided). Although not statistically significant, the average time in the experimental group was shorter than the control group (175 s vs. 401 s). No clinical/significant differences were found in pain, patient satisfaction, or the subjective difficulty for the healthcare professional.	The use of ultrasound for refilling can decrease needle-in-skin time, the number of punctures, changes in needle plane, and the need for intervention from other professionals.
Pinho et al., 2022 [15]	1 case report with 1 patient	To demonstrate that the use of ultrasound in difficult refill cases can simplify the technique and prevent complications.	Ultrasound was used for a refill in a complicated case with difficult access. The indirect method was used (the area was marked by visualizing the refill port) before puncturing. In the end, it was confirmed that there was no fluid in the subcutaneous pocket.	The use of ultrasound is safe and simple for locating the refill port of intrathecal infusion pumps. It can facilitate the procedure and prevent complications. It can be useful for pumps implanted more than 10 mm from the skin.
Singa et al., 2020 [18]	Randomized controlled trial. 107 patients. Period of 11 months.	To identify the outcomes obtained with the traditional method vs. ultrasound for refilling. To assess patient preferences.	192 refills were performed (67 with a template and 125 with ultrasound). 84% of those refilled with ultrasound reported no pain, compared to 67% of those refilled with a template. The median (quartile) duration of the port access procedure was 60 (35 to 109) seconds with the use of a template compared to 90 (66 to 122) seconds following ultrasound use. Most patients prefer the use of ultrasound for refilling.	Patients prefer refilling with ultrasound because they feel it is less painful than the traditional method. They report that ultrasound refills take longer than the traditional (template) method.
Matthys et al., 2020 [17]	Randomized controlled trial. 22 patients. Period of 10 months	To identify situations where the use of ultrasound is more precise than the traditional template method.	81 refills were performed with ultrasound on 22 patients. No correlation was observed between age, height, weight, body mass, and abdominal perimeter during refilling. Better results with ultrasound for pumps implanted more than 10 mm deep (Spearman rho = 0.697, *p* < 0.001).	The use of ultrasound increases the safety of refilling for pumps implanted more than 10 mm deep. For devices implanted more superficially (less than 10 mm), the template is as accurate as ultrasound. It is crucial to use the most appropriate method based on the pump’s depth to prevent complications.
Maino et al., 2018 [16]	Randomized controlled trial. 19 patients. Period of 24 months.	To evaluate whether access to the refill port in intrathecal infusion pumps is easier using ultrasound compared to the traditional “blind” method	111 refills were performed on 19 patients over a 24-month period. They suggest that the blind technique requires fewer attempts than the use of ultrasound (*p* = 0.018). Ultrasound requires more time for refilling (*p* = 0.001). There were no significant differences in the number of punctures or perceived pain.	The results lead them to believe that fewer attempts are required with the traditional method than with the use of ultrasound. Ultrasound can be useful in complex cases.
García Eslava et al., 2018 [14]	1 case report with 1 patient	To demonstrate that the use of ultrasound in refilling intrathecal infusion pumps improves the technique.	Ultrasound was used for a refill on a patient with difficult port access. The number of punctures was reduced, and patient satisfaction was increased	The risks associated with refilling are reduced. The learning time for the technique can be less than 30 min.
Caruso et al., 2018 [13]	Descriptive study of 2 cases with 2 patients	To report experience with the use of ultrasound for baclofen intrathecal infusion pump refills. To describe cases with complications after implant. To suggest a clinical method for ultrasound-guided refilling.	They report that the use of ultrasound increases the safety of the technique by reducing pain and the risk of infection. It improves the precision of the procedure.	The use of ultrasound can improve the intrathecal pump refill technique in difficult cases, although more studies are needed.
Maneyapanda et al., 2016 [12]	3 case reports with 3 patients	To demonstrate the usefulness of ultrasound for refilling intrathecal infusion pumps in patients with difficult port access. To review the literature on the use of ultrasound for intrathecal infusion pump refills.	They present 3 cases of intrathecal infusion pump refills with ultrasound. They review the literature on possible complications derived from the technique.	The use of ultrasound is expected to prevent some complications of refilling.
Saulino et al., 2014 [11]	Descriptive study of intrathecal infusion pumps	To demonstrate the unique sonographic characteristics of intrathecal infusion pumps.	Intrathecal infusion pumps were analyzed outside the human body. They describe the sonographic characteristics of the pumps, highlighting that the refill port is anechoic, in contrast to the pump’s surface, which is hyperechoic.	The programmable intrathecal infusion systems have a unique sonographic appearance that could be used for refilling them.
Peccora et al., 2013 [10]	1 case report with 1 patient	To describe how ultrasound can help detect extravasation in the subcutaneous pocket.	The use of ultrasound for the detection of extravasation (outside the infusion pump) in a patient is explained.	Ultrasound could be used as a rapid and effective means of detecting extravasation in the subcutaneous pocket of intrathecal infusion pumps.
Gofeld et al., 2011 [9]	Preclinical feasibility study. 1 case report with 1 patient (cadaver).	To describe the methodology for real-time ultrasound-guided refill of intrathecal infusion pumps.	The learning curve for inexperienced users indicates low complexity of the technique. The predictive value of the needle is 100%.	The use of ultrasound for refilling intrathecal pumps is feasible and safe, and it can improve their care and maintenance. Complications such as subcutaneous injections can be prevented.
Shankar et al., 2009 [8]	1 case report of 1 patient	To demonstrate that the use of ultrasound can assist in detecting the refill port in difficult cases.	The patient presented with a seroma, making access to the refill port difficult. Ultrasound was used, which facilitated the performance of the technique.	This is a successful demonstration of the use of ultrasound for detecting the refill port in a difficult case. It avoids multiple punctures and possible complications. It proves to be a safe technique in complex cases.
Hurdle et al., 2007 [7]	1 case report with 1 patient	To demonstrate that the use of ultrasound for refilling intrathecal infusion pumps can facilitate access and prevent complications.	A case of a person with difficult access to the refill port due to degenerative kyphoscoliosis and increased body mass was studied.	The use of ultrasound proves to be a simple and effective technique. There are no reliable data on the reduction in time spent on refilling.

## Data Availability

Data regarding this study is available upon request to the corresponding author.
